# Supplementary material for: Alterations in homologous recombination repair genes in prostate cancer brain metastases
Source: Nat Commun. 2022 May 3;13:2400. doi: 10.1038/s41467-022-30003-5 (PMC9065149; doi:10.1038/s41467-022-30003-5)
Supplement: Supplementary file 1 — Supplementary Information [file 41467_2022_30003_MOESM1_ESM.pdf]

Supplementary Information

Alterations in Homologous Recombination Repair Genes in Prostate Cancer Brain Metastases

Antonio Rodriguez-Calero[1,2 §], John Gallon[3 §], Dilara Akhoundova[1,4], Sina Maletti[1], Alison Ferguson[1,5], Joanna Cyrta[6], Ursula Amstutz[7], Andrea Garofoli[8], Viola Paradiso[8], Scott A. Tomlins[9], Ekkehard Hewer[2,10], Vera Genitsch[2], Achim Fleischmann[2,11], Erik Vassella[2], Elisabeth J. Rushing[12], Rainer Grobholz[13], Ingeborg Fischer[13], Wolfram Jochum[14], Gieri Cathomas[15], Adeboye O. Osunkoya[16], Lukas Bubendorf[8], Holger Moch[17], George Thalmann[18], Charlotte K.Y. Ng[1], Silke Gillessen[19,20,21#], Salvatore Piscuoglio[3,8 #*], and Mark A. Rubin[1,22 #*]

[*] these authors contributed equally (first co-authorship)
[#] senior co-authorship
[*] Correspondence: s.piscuoglio@unibas.ch and mark.rubin@dbmr.unibe.ch

1 Department for BioMedical Research, University of Bern, Bern, Switzerland
2 Institute of Pathology, University of Bern, Bern, Switzerland
3 Visceral Surgery and Precision Medicine Research Laboratory, Department of Biomedicine, University of Basel, Basel, Switzerland
4 Department of Medical Oncology and Hematology, University Hospital Zurich, Zurich, Switzerland
5 Department of Oncology, Ludwig Cancer Centre, University of Lausanne, Lausanne, Switzerland
6 Department of Pathology, Institut Curie, University Paris Sciences et Lettres, Paris, France
7 Department of Clinical Chemistry, Inselspital, Bern University Hospital, University of Bern, Bern, Switzerland
8 Institute of Medical Genetics and Pathology, University Hospital Basel, University of Basel, Switzerland
9 Departments of Pathology and Urology, University of Michigan Medical School, Ann Arbor, Michigan, USA
10 Institute of Pathology, Lausanne University Hospital and University of Lausanne, Lausanne, Switzerland (current address)
11 Institute of Pathology, Cantonal Hospital Thurgau, Münsterlingen, Switzerland
12 Institute of Neuropathology, University Hospital Zurich, Zurich, Switzerland
13 Institute of Pathology, Cantonal Hospital Aarau, Aarau, Switzerland
14 Institute of Pathology, Cantonal Hospital St. Gallen, St. Gallen, Switzerland
15 Institute of Pathology, Cantonal Hospital Baselland, Liestal, Switzerland
16 Departments of Pathology and Laboratory Medicine, and Urology, Emory University School of Medicine, Atlanta, USA
17 Department of Pathology and Molecular Pathology, University Hospital Zurich, Zurich, Switzerland
18 Department of Urology, Inselspital, Bern University Hospital, Bern, Switzerland
19 Faculty of Biomedical Sciences, USI, Lugano
20 Department of Oncology, Cantonal Hospital St. Gallen, St. Gallen, Switzerland
21 Division of Cancer Sciences, University of Manchester, Manchester, United Kingdom
22 Bern Center for Precision Medicine, Inselspital, Bern University Hospital, University of Bern, Switzerland

Supplementary Figure 1

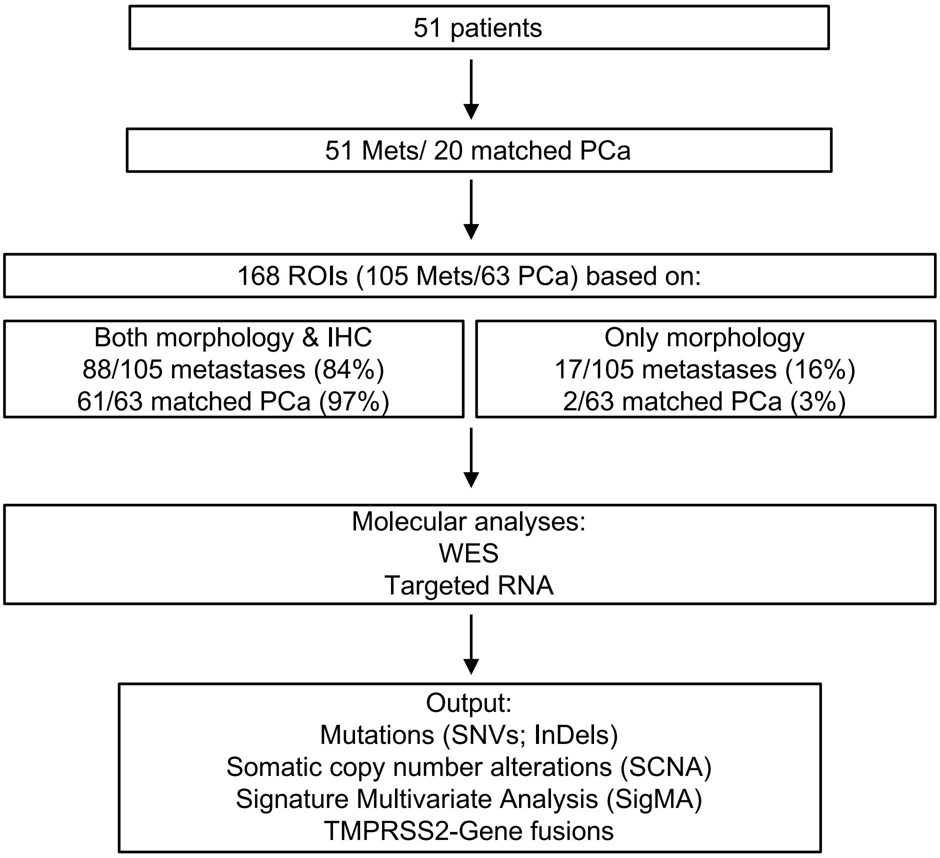

PCBM cohort establishment and conducted methods. For 20 of the 51 patients included, we had both metastatic and matched primary samples. A total of 168 intratumoral regions of interest (ROIs) were selected based on morphology and, whenever enough tissue was available, additional immunohistochemistry. Each defined ROI within metastases (105 ROIs) and primary tumors (63 ROIs) represents one sample of this cohort. All samples underwent whole exome sequencing (WES) and targeted RNA analyses.

Supplementary Figure 2

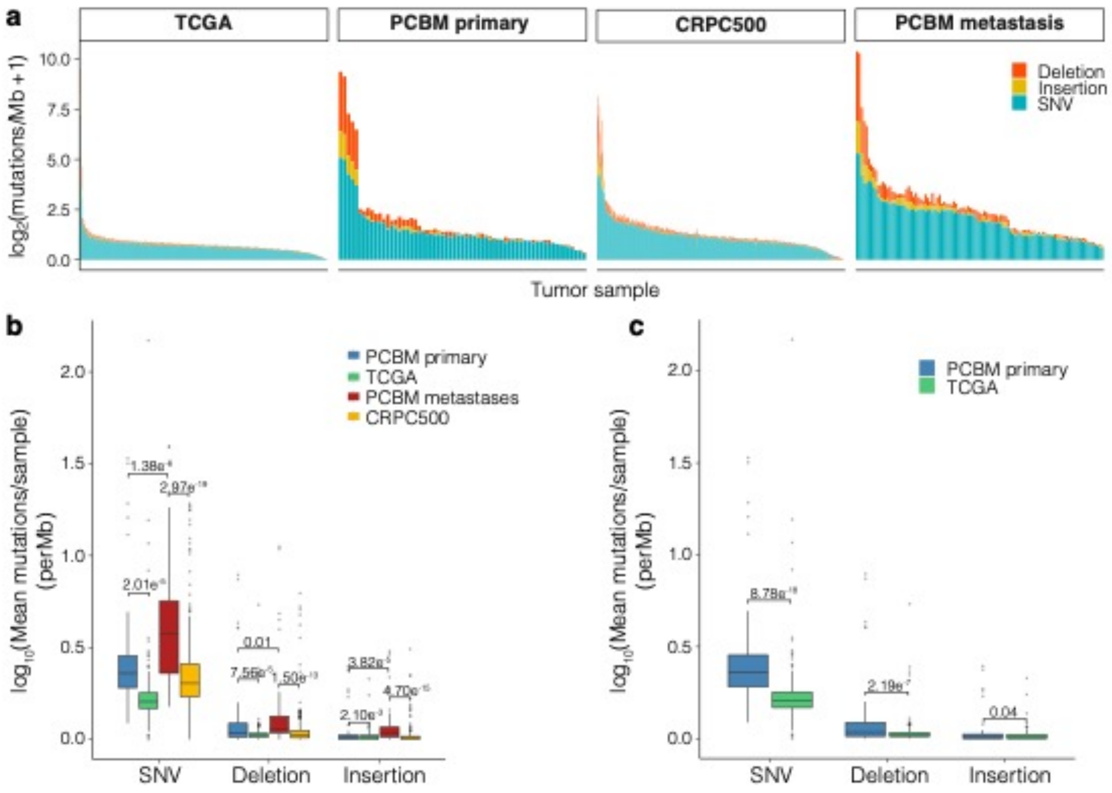

Mutational burden in PCBM cohort. a. Summary of tumor mutational burden for SNVs, insertions and deletions in PCBM primary, TCGA PCa primary, CRPC500 metastatic, and PCBM metastatic samples. **b**. Mean mutational burden for samples as from **a**. **c.** As in **b** but comparing only PCa primary with high grade (GG >3) TCGA samples. PCa n = 63, TCGA n = 495, TCGA GG>3 n= 286, PCBM n = 105, CRPC500 = 431. Horizontal lines in boxplots show mean, hinges show interquartile range, whiskers show 1.5 x interquartile range, points beyond 1.5 x IQR past hinge are shown Two-sided Wilcox test, FDR corrected *P* values. Source data are provided as a Source Data file.

Supplementary Figure 3

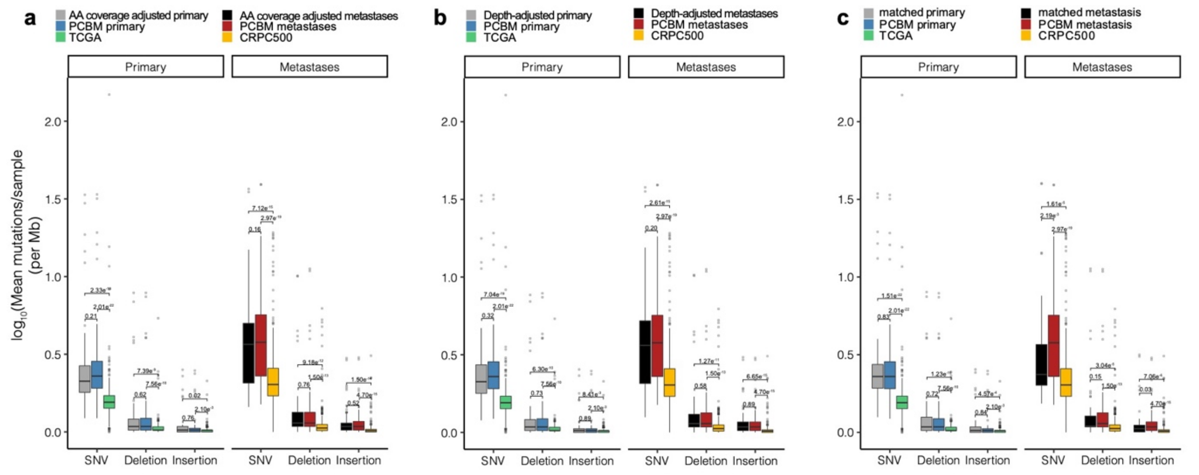

Differences in TMB are robust to technical differences across cohorts, and the absence of matched normal for a subset of PCBM cases. a) Mean TMB in PCBM samples, with and without adjusting for depth by increasing threshold of # supporting reads 1.6 fold above the threshold used in the CRPC500 i.e. 16 reads. PCBM samples are compared to the CRPC500 cohort (metastases) or TCGA (primary PCa) PCBM primary n = 63, TCGA n = 495, PCBM metastases n = 105, CRPC500 = 431. **b**) Mean TMB in PCBM samples, with and without adjusting for depth by increasing thresholds for VAF and # supporting reads 2.58-fold, compared to the CRPC500 cohort (metastases) or TCGA (primary PCa). PCBM primary n = 63, TCGA n = 495, PCBM metastases n = 105, CRPC500 = 431. **c**) Mean TMB in PCBM samples (whole cohort), or only those for which matched normal tissue was available, compared to the TCGA (PCa) or CRPC500 cohort (metastases). PCBM primary n = 63, matched primary n = 61, TCGA n = 495, PCBM metastases n = 105, matched metastases n = 62, CRPC500 n = 431). Horizontal lines in boxplots show mean, hinges show interquartile range, whiskers show 1.5 x interquartile range, points beyond 1.5 x IQR past hinge are shown. Two-sided Wilcoxon test, FDR adjusted *P* values.

Supplementary Figure 4

a

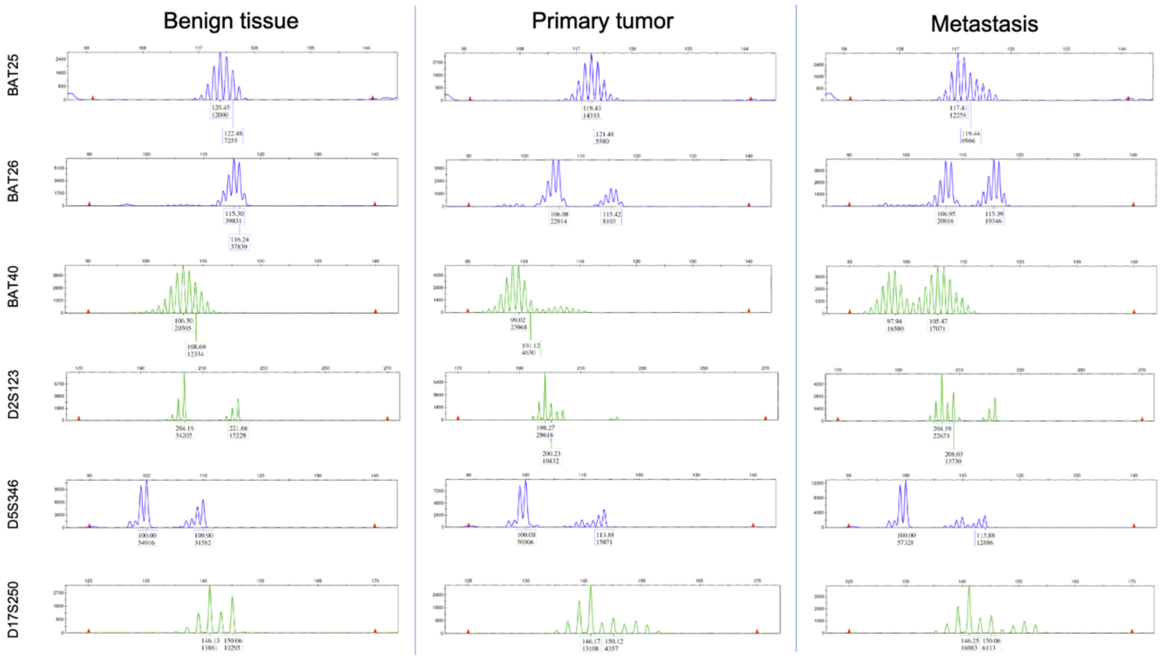

b

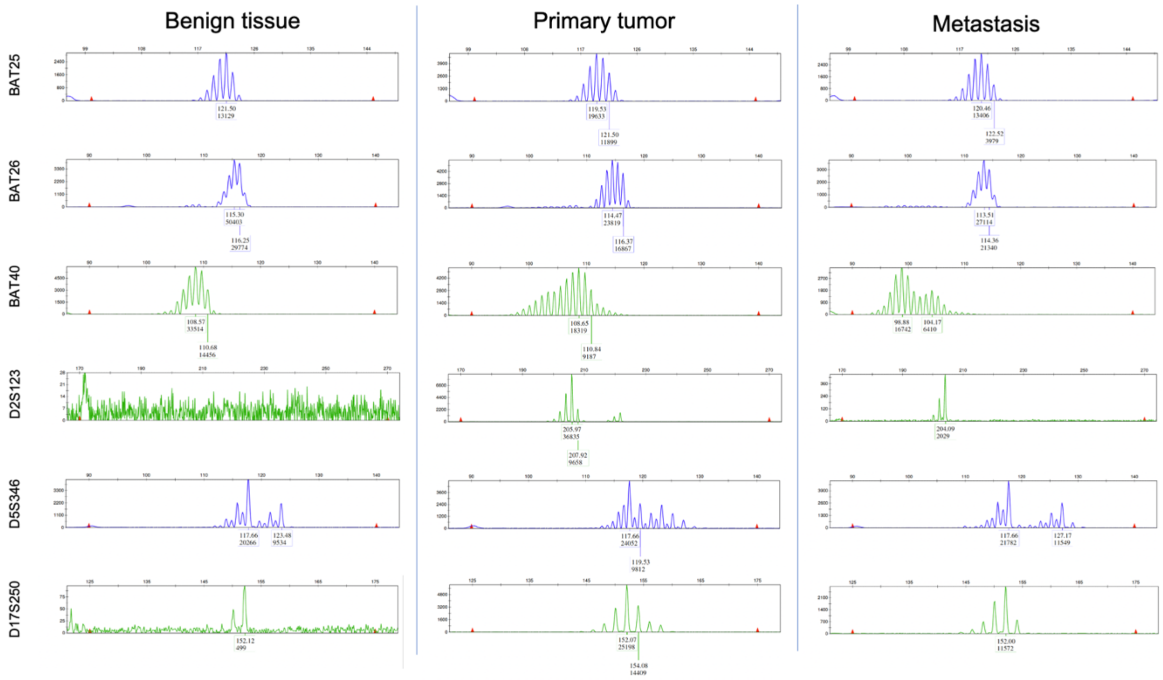

Microsatellite instability. MSI was assessed for patient 39 (a) and 48 (b) from primary tumor, brain metastasis and matched benign tissue by using five microsatellite loci recommended by the Bethesda panel plus BAT40. PCR products were analyzed by capillary electrophoresis using a 3500 genetic analyzer (Applied Biosystems). Both patients showed high microsatellite instability (MSI-H) status in the tumor components, primary tumor and metastasis, compared with the matched benign tissue.

Supplementary Figure 5

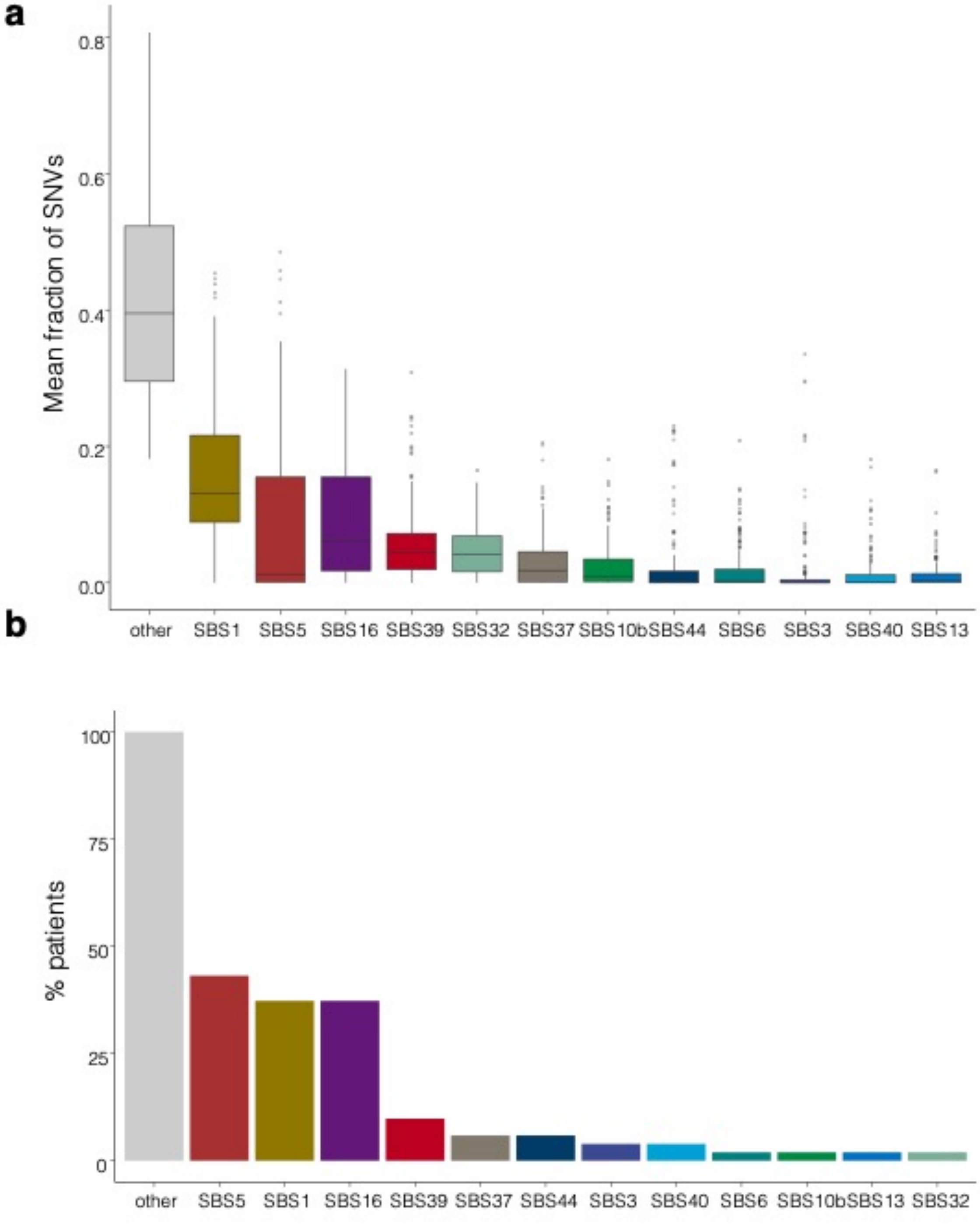

Mutational signatures present in the PCBM cohort. a. Relative contribution of mutational signatures in the PCBM cohort, ordered by mean contribution. Signatures with relative contribution > 0.15 in at least one sample are shown. Signatures were calculated as the average from deconstructSigs and Mutational Patterns. Horizontal lines in boxplots show median, hinges show interquartile range, whiskers show 1.5 x interquartile range, points beyond 1.5 x IQR past hinge are shown **b.** Percentage of patients in which each signature was detected. Signatures with relative contribution > 0.15 in at least one sample are shown. N=51 patients. Source data are provided as a Source Data file.

Supplementary Figure 6

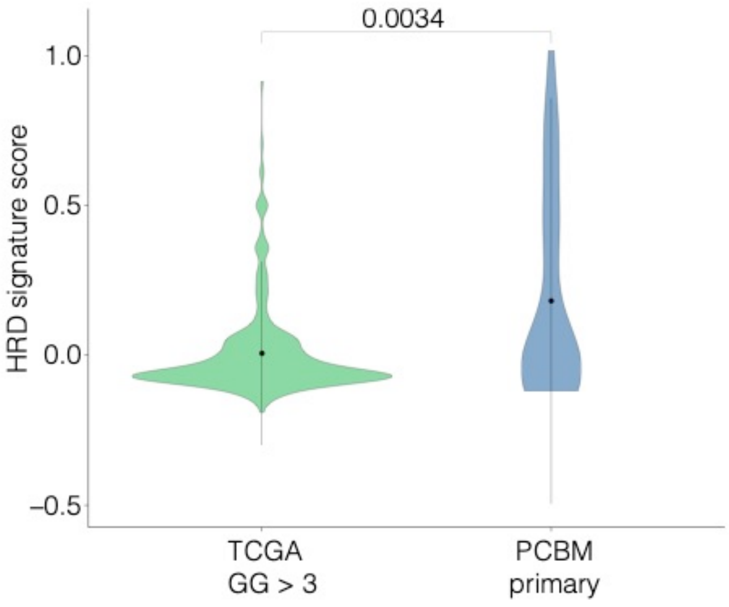

Comparison of SBS3 signature score in PCa primary samples from the PCBM cohort and high grade TCGA samples. Points show mean, lines show data range. TCGA n = 286, PCa n = 20. Source data are provided as a Source Data file.

Supplementary Figure 7

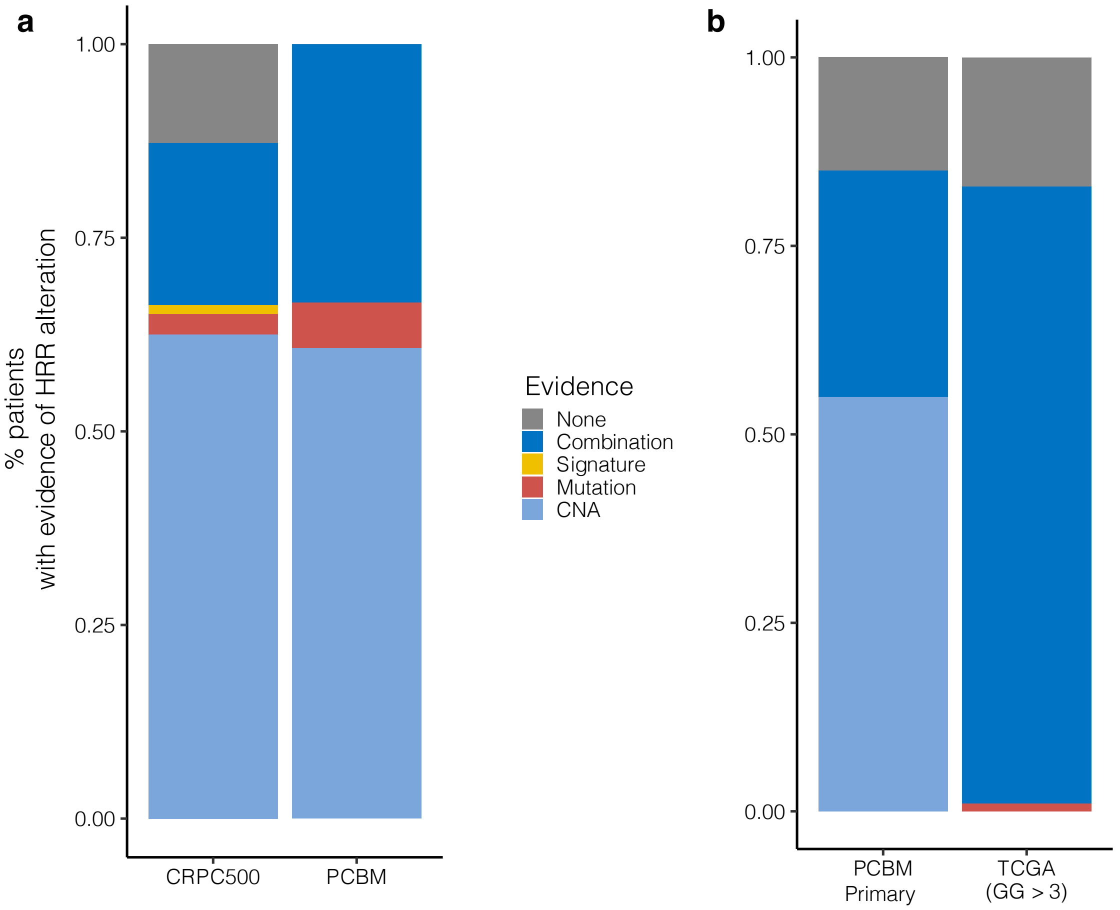

Frequency of HRR gene alterations and presence of SBS3 across cohorts. a. PCBM and CRPC500 non-brain metastases. PCBM n = 51, CRPC500 n = 416. **b.** Primaries from PCBM cohort and TCGA high grade samples (PCa n=20, TGCA n=281). Source data are provided as a Source Data file and in Supplementary Table 4 and 5.

Supplementary Figure 8

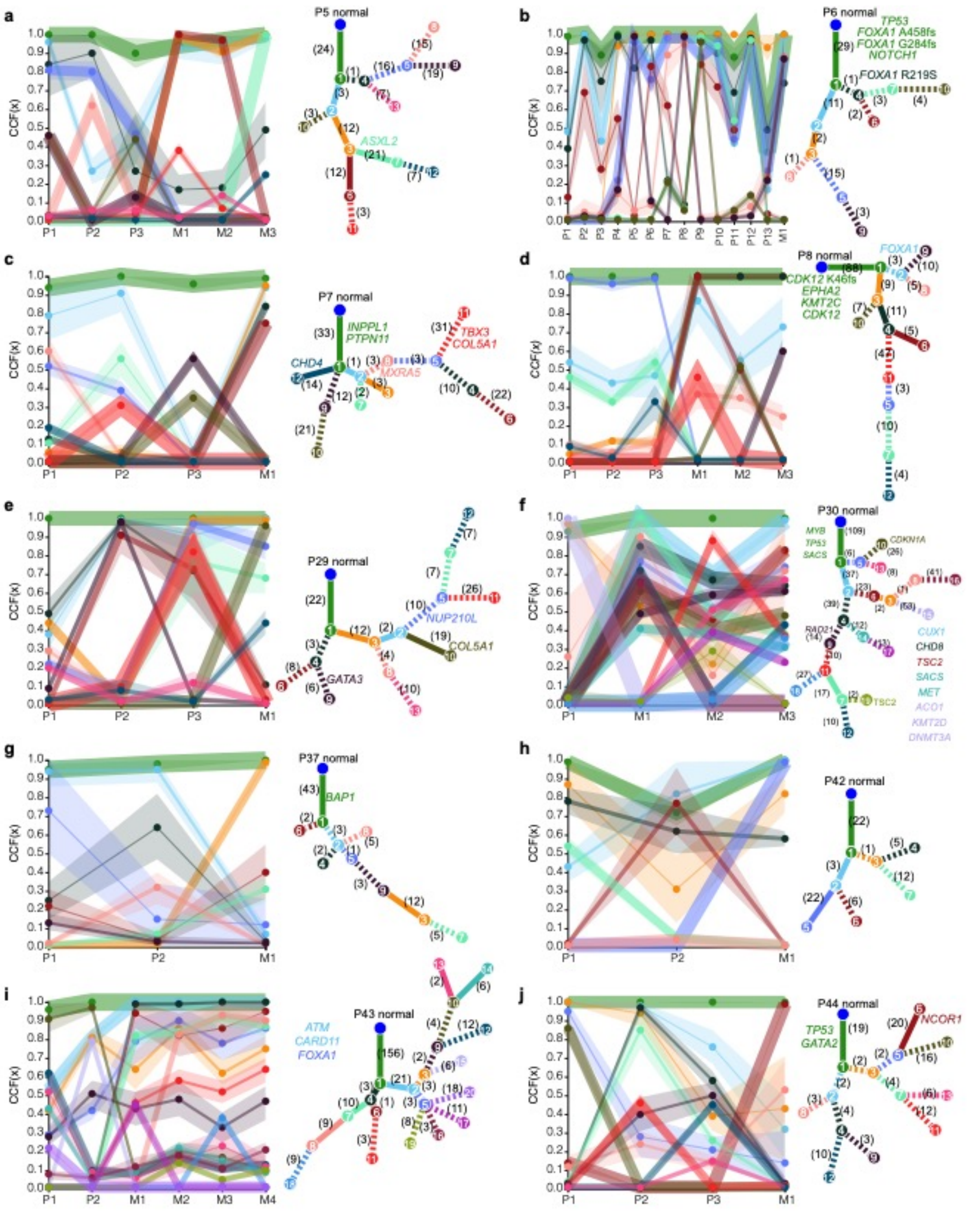

Clonal evolution in ten PCBM cases with matched primary and metastatic samples. Trace plots show cancer cell fraction (CCF) for each mutational cluster in each sample from each patient **a-j** (P = primary, M = metastasis). Ribbons show 95 % confidence interval, centre of bands show mean cluster CCF estimate. Phylogenetic trees show best solution for evolutionary relationship between clones with different clusters of mutations where each node (numbered) is a cluster of mutations. Numbers on each branch show the number of mutations distinguishing a clone from the previous (all genes). Potential driver genes mutated in the distinction between a clone and the previous are indicated in colours corresponding to the branch. Solid branches show clusters of mutations which become clonal in metastatic samples. Source data are provided as a Source Data file.

Supplementary Figure 9

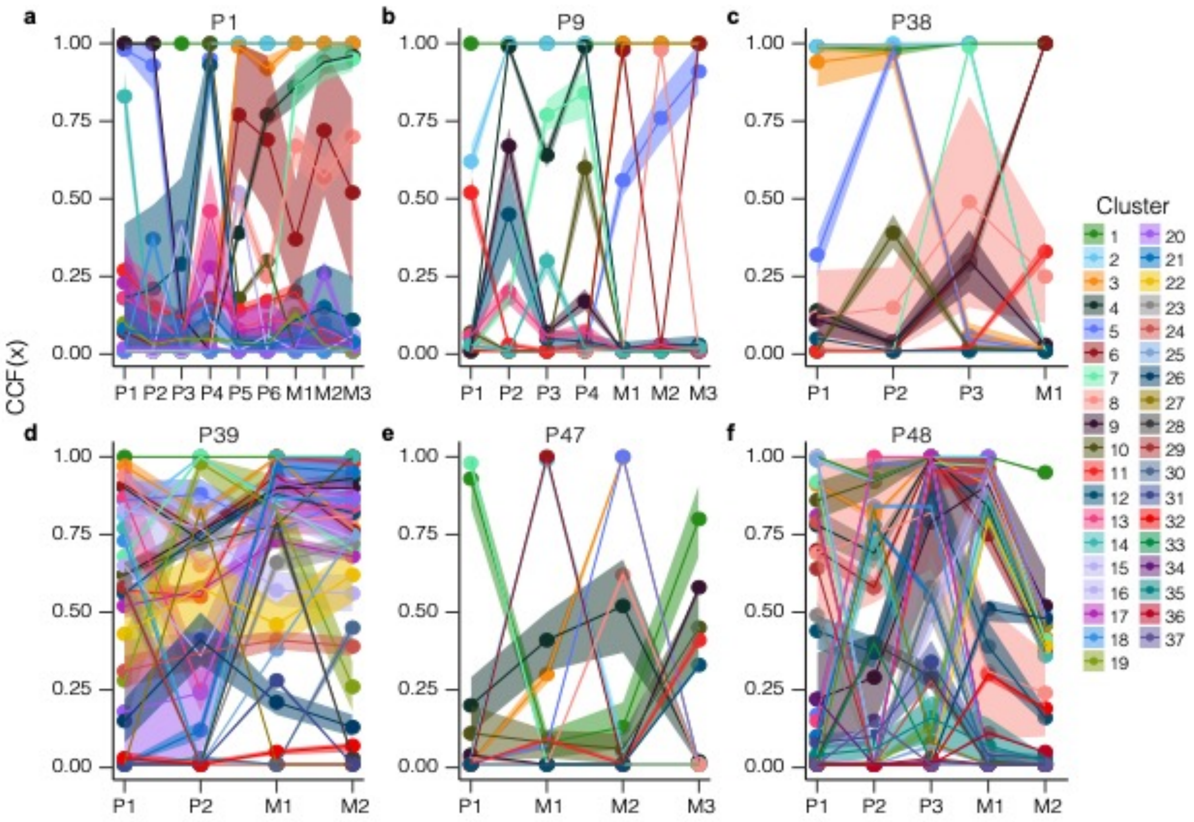

Cancer cell fraction of mutation clusters in six PCBM cases for which tree solutions could not be calculated. Trace plots **a-f** show CCF for each mutational cluster in each patient sample (P = primary, M = metastasis). Ribbons show 95 % confidence interval, centre of bands shows mean cluster CCF estimate. Source data are provided as a Source Data file.

Supplementary Figure 10

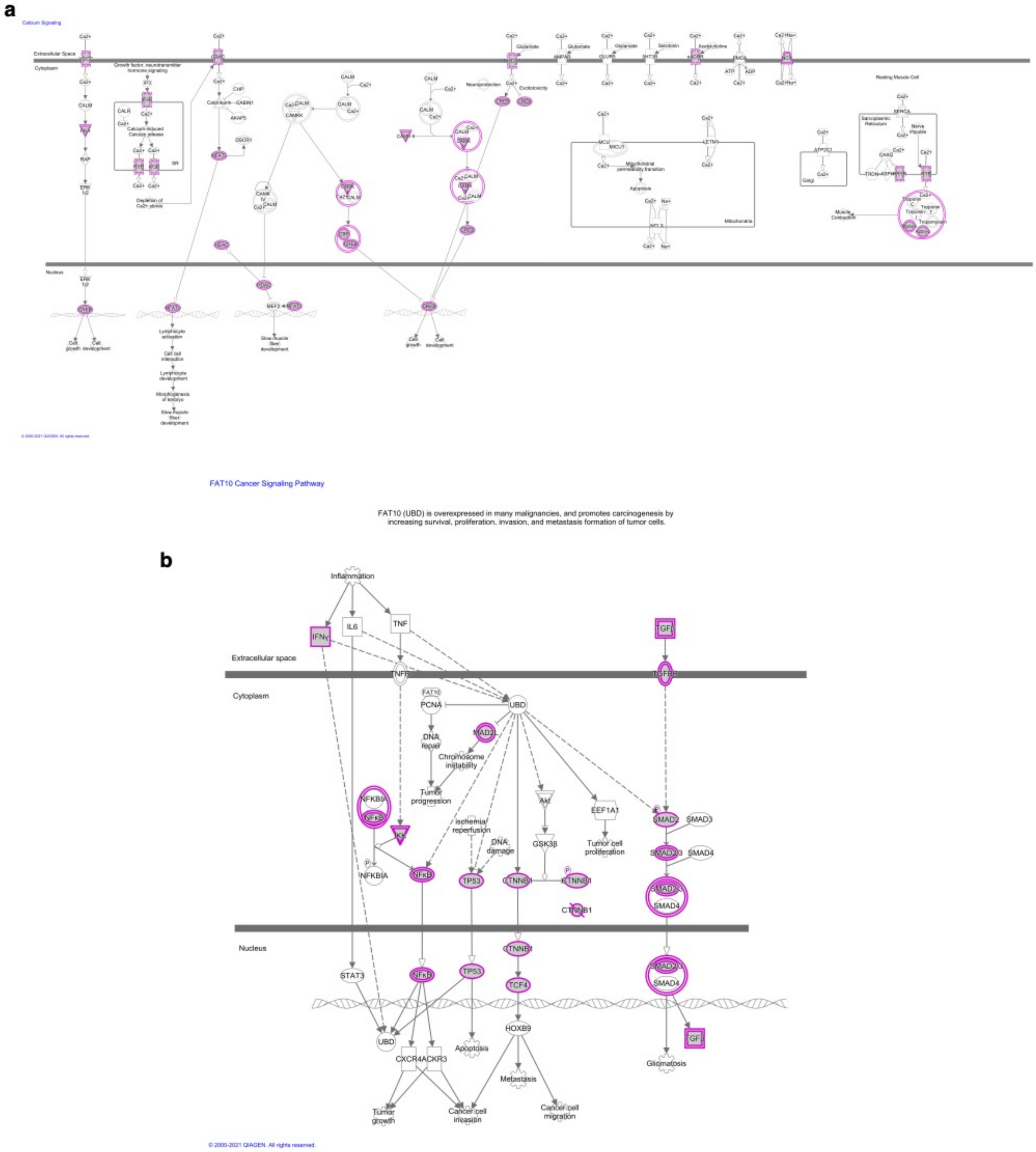

Canonical pathways enriched for alterations in metastatic-clonal clusters. Diagrams from Ingenuity Pathway Analysis showing canonical pathways (**a**) calcium signaling and (**b**) FAT10 signaling enriched for metastatic clonal mutations in PCBM. Altered molecules are highlighted in pink.

Supplementary Table 1. Demographic data and analysed tissue

Patient-N	Research ID	Primary tumor available	Number CNS metastases	Location	In this study included type	Additional non-brain metastases	ADT or orchidectomy	Abiraterone	Enzalutamide
1	19.1	YES	Singular	Fronto-parietal right	Dura	Bone	Yes	No	No
4	19.4	YES	Singular	Frontal right	Dura	Bone	Yes	No	No
5	19.5	YES	Multiple	Cerebellar	Brain	Bone	Yes	No	No
6	19.6	YES	Singular	Cerebellar right	Brain	Bone	Yes	No	No
7	19.7	YES	Multiple	Occipital left	Dura with brain involvement	Bone	Yes	No	No
8	19.8	YES	Multiple	Cerebral, NOS	Brain	Bone	Yes	No	No
9	19.9	YES	Unknown	NA	Dura	Unknown	Yes	No	No
10	19.10	NO	Singular	Cerebellar	Brain	Bone (spine, pelvis)	Yes	No	No
12	19.12	NO	Singular	Cerebellar right	Brain	Unknown	Yes	No	No
14	19.14	YES	Multiple	Temporal	Dura	Bone (spine, pelvis)	Yes	No	No
16	19.16	YES	Singular	Temporal right	Dura with brain involvement	Bone, Lymph nodes	Yes	Yes	No
17	19.17	NO	Multiple	Cerebral, NOS	Brain	Bone	Yes	Yes	No
18	19.18	NO	Multiple	Cerebral, NOS	Brain	Bone (multiple)	Yes	Yes	No
19	19.19	NO	Singular	Left temporal	Brain	Unknown	Yes	No	No
20	19.20	NO	Singular	Left temporal	Unknown	Bone	Yes	No	No
21	19.21	NO	Singular	Left frontal	Brain	None	No	No	No
22	19.22	NO	Singular	Cerebellar right	Brain	Bone, Lymph nodes	Yes	No	No
23	19.23	NO	Multiple	Temporal bilateral	Brain	Bone, Liver	Yes	No	No
24	19.24	NO	Multiple	Infraselar	Dura	Bone, Lung	Yes	No	No
25	19.25	NO	Singular	Frontal left	Dura	Bone, Lung	Yes	No	No
26	19.26	NO	Singular	Parietal left	Brain	Metastasized, unknown locations	Yes	No	No
27	19.27	NO	Multiple	NA	Dura	Bone, Lymph nodes	Yes	Yes	No
29	19.29	YES	Singular	Cerebellum	Unknown	Bone, Lymph nodes	Yes	No	No
30	19.30	YES	Multiple	Frontal bilateral	Dura	Bone, Lung	Yes	No	No
31	19.31	NO	Singular	Cerebellar left	Dura	Bone	No	Unknown	Unknown
32	19.32	YES	Multiple	Parietal/occipital right; temporal left	Dura	Bone	Yes	No	No
33	19.33	NO	Multiple	Thoracic spine 4-6	Dura	Bone	No	No	No
34	19.35	NO	Multiple	Paracentral left	Brain	Lung, Lymph nodes	Yes	Yes	Yes
35	19.36	NO	Multiple	Temporal bilateral, frontal left	Dura with brain involvement	Bone, Lymph nodes, Liver, Lung	Yes	No	No
36	19.37	NO	Multiple	NA	Brain	Bone, Lymph nodes	No	No	No
37	19.50	YES	Singular	Fronto-basal left	Brain with dura/bone involvement	Unknown	Unknown	No	No
38	19.51	YES	Multiple	Cerebellum	Brain	Lymph nodes mediastinal	Unknown	No	No
39	19.52	YES	Singular	Cerebellum/ ventricle IV	Brain	None	No	No	No
40	19.53	NO	Singular	Parietal right	Dura/Bone	Bone	No	Yes	No
42	19.40	YES	Multiple	Precentral gyrus right	Brain	Bone, Lung, Pleura, Lymph node	Yes	Yes	No
43	19.41	YES	Multiple	Parietal bilateral	Dura	Bone, Liver, Lymph node	Yes	Yes	No
44	19.42	YES	Multiple	Frontal, Temporal, Occipital, Cerebellum, Meninge	Brain	Bone, Lung, Liver, Lymph node	Yes	No	No
45	19.43	YES	Multiple	Parietal left, Brain stem, C1-C7, Lumbar, Sacral	Dura	Bone, Liver, Muscle	Yes	No	No
46	19.44	NO	Multiple	Unknown	Dura	Bone, Lung, Liver, Spleen, Adrenal gland, Kidney	Yes	No	No
47	19.39	YES	Singular	Frontal right	Brain	Lymph node	Yes	No	No
48	19.48	YES	Multiple	Cerebellum	Brain	Bone, Lymph node, Adrenal gland, Soft tissue	Yes	No	No
49	19.49	YES	Multiple	Frontal left	Dura	Bone, Lung, Pleura, Muscle	Yes	No	No
50	19.64	NO	Singular	Left parietal, NOS	Brain/Dura	Bone	Yes	No	No
51	19.65	NO	Singular	Frontal right	Brain/Dura	Bone, Lung, Liver, Lymph node	No	No	No
52	19.66	NO	Multiple	Spinal cord/L5	Cord/Dura/Bone	Bone, Lung, Lymph node	Yes	Yes	Yes
53	19.67	NO	Multiple	Parietal right	Brain/Dura	Bone, Bladder (direct extension), Lymph node	Yes	No	No
54	19.68	NO	Multiple	Spinal cord thoracic, NOS	Cord/Dura/Bone	Bone	Yes	Yes	No
55	19.69	NO	Multiple	Spinal cord, NOS	Cord/Dura/Bone	Bone, Lymph node	Yes	No	No
57	19.71	NO	Singular	Spinal cord thoracic, NOS	Cord/Dura/Bone	Bone, Lymph node	Yes	No	Yes
58	19.72	NO	Multiple	Spinal cord T3	Cord/Dura/Bone	Bone	Yes	Yes	Yes
59	19.73	NO	Multiple	Spinal cord thoracic, NOS	Cord/Dura/Bone	Bone, Liver, Lymph node	Yes	No	No

Supplementary Table 2. Summary of large cohorts including metastases of prostate cancer within the last 10 years

	Publication	N° Samples of metastatic prostate cancer		N° Patients of metastatic prostate cancer		Methods
		All locations	PCBM	PCBM	Matched primary to PCBM	
1	Taylor et al. Cancer Cell. 2010	37	12	12	No	Targeted DNA/RNA-seq/miRNA expression
2	Grasso et al. Nature. 2012	50	1	1	No	Targeted-DNA/RNA-seq
3	Gundem et al. Nature. 2015	53	3	2	1	WGS
4	Beltran et al. Nature Med. 2016	112*	4	3	No	WES/RNA-seq/Targeted RNA
5	Kumar et al. Nature Med. 2016	154	0	0	n/a	WES/RNA-seq
6	Abida et al. JCO Precis Oncol. 2017	228	0	0	n/a	Targeted DNA
7	Aggarwal et al. J Clin Oncol. 2018	249	0	0	n/a	RNA-seq, Targeted DNA
8	Abida et al. PNAS. 2019	444**	1	1	No	WES/RNA-seq
9	Van Dessel et al. Nat Commun. 2019	197	0	0	n/a	WGS
10	Mateo et al. J Clin Invest. 2019	61	0	0	n/a	Low pass WES/Targeted-DNA
		**1585**	**21**	**19**	**1**	
**11**	**Current study**	**105**	**105**	**51**	**20**	**WES/Targeted RNA**

Ten large cohorts including molecular analyses of metastatic prostate cancer were reviewed based on available data from cBioPortal or publications (1-10). Overlapping patients were excluded (top). Patients and samples investigated in the current study (bottom). PCBM: including metastases from following locations: brain, dura (also epidural, subdural) and spinal cord. * 2 patients were previously published in Baca et al, Cell, 2013 (11). ** 150 patients were previously published in Robinson et al, Cell, 2015 (12).

References

1. Taylor, B.S. et al. Integrative genomic profiling of human prostate cancer. Cancer Cell 18, 11-22 (2010).
2. Grasso, C.S. et al. The mutational landscape of lethal castration-resistant prostate cancer. Nature 487, 239-43 (2012).
3. Gundem, G. et al. The evolutionary history of lethal metastatic prostate cancer. Nature 520, 353-357 (2015).
4. Kumar, A. et al. Substantial interindividual and limited intraindividual genomic diversity among tumors from men with metastatic prostate cancer. Nat Med 22, 369-78 (2016).
5. Aggarwal, R. et al. Clinical and Genomic Characterization of Treatment-Emergent Small-Cell Neuroendocrine Prostate Cancer: A Multi-institutional Prospective Study. J Clin Oncol 36, 2492-2503 (2018).
6. Abida, W. et al. Genomic correlates of clinical outcome in advanced prostate cancer. Proc Natl Acad Sci U S A (2019).
7. Beltran, H. et al. Divergent clonal evolution of castration-resistant neuroendocrine prostate cancer. Nat Med 22, 298-305 (2016).
8. Abida, W. et al. Prospective Genomic Profiling of Prostate Cancer Across Disease States Reveals Germline and Somatic Alterations That May Affect Clinical Decision Making. JCO Precis Oncol 2017(2017).
9. van Dessel, L.F. et al. The genomic landscape of metastatic castration-resistant prostate cancers reveals multiple distinct genotypes with potential clinical impact. Nat Commun 10, 5251 (2019).
10. Mateo, J. et al. Genomics of lethal prostate cancer at diagnosis and castration resistance. J Clin Invest 130, 1743-1751 (2020).
11. Baca, S.C. et al. Punctuated evolution of prostate cancer genomes. Cell 153, 666-77 (2013).
12. Robinson, D. et al. Integrative clinical genomics of advanced prostate cancer. Cell 161, 1215-1228 (2015).

Supplementary Table 3. Patients fulfilling PROfound criteria*

Patients with homozygous deletion	Gen					
57	PPP2R2A					
27	BRCA2					
30	BRCA2					
45	PPP2R2A					
47	PPP2R2A, CHEK2, RAD54L, BRCA1					
12	PPP2R2A					
**Patients with truncating mutations**	**Gen**	**Protein_Change**	**Variant_Classification**	**Clonal Status**	**ClinVar**	**Other sources**
39	BRCA1	p.Lys339fs	Frame_Shift_Del	clonal	National Center for Biotechnology Information. ClinVar; [VCV000266130.2], https://www.ncbi.nlm.nih.gov/clinvar/variation/VCV000266130.2 (accessed Feb. 26, 2022).	
48	BRIP1	p.Leu54*	Nonsense_Mutation	clonal	National Center for Biotechnology Information. ClinVar; [VCV000935760.2], https://www.ncbi.nlm.nih.gov/clinvar/variation/VCV000935760.2 (accessed Feb. 26, 2022).	
1	BRCA2	p.Arg3005*	Nonsense_Mutation	clonal	National Center for Biotechnology Information. ClinVar; [VCV000234445.8], https://www.ncbi.nlm.nih.gov/clinvar/variation/VCV000234445.8 (accessed Feb. 26, 2022).	
8	CDK12	p.Gln612*	Nonsense_Mutation	subclonal	Not reported	PMID: 33804295-Breast Cancer
	CDK12	p.Lys46fs	Frame_Shift_Del	clonal	Not reported	
**Total of patients: 10/51= 19.6%**						

According to the Profound criteria:** ***A patient had a qualifying alteration if any deleterious or suspected deleterious alteration was found in the 15 pre-specified genes with a direct or indirect role in HRR. An alteration was regarded as deleterious if it results in protein truncation (which includes nonsense, frameshift, or consensus splice site alterations), or select missense alterations well-known to be deleterious in ClinVar/BIC databases. Furthermore, larger- scale alterations, such as genomic truncating rearrangements or homozygous deletions, were also classified as qualifying.

Supplementary Table 4. Summary of the protocols used for DNA & RNA extraction, library preparation and sequencing for the different methods

Method	Reagent Kit	Supplier	Protocol	Page numbers	Comment
**6.1. Nucleic acid isolation**					
6.1.1. FFPE samples	AllPrep DNA/RNA FFPE Kit	Qiagen	AllPrep FFPE DNA RNA		Protocol for DNA and RNA isolation from one sample, used for targeted sequencing
	QIAamp DNA Micro Kit	Qiagen	DNA Extraktion mit MicroKit		DNA extraction used for WES (protocol from CGL)
6.1.2. Tissue or blood samples, cells	DNeasy Blood and Tissue Kit	Qiagen	DNeasy Blood and Tissue Handbook	25 – 30	Protocol for DNA isolation
**6.2. Sequencing**					
6.2.1. Targeted RNA sequencing					Sequencing: Ion Chef and S5
cDNA synthesis	SuperScript VILO	Thermofisher	Ion Ampliseq Library Kit plus User Guide	72	
Library preparation	Ion Ampliseq Library Kit plus, Xpresss barcodes	Thermofisher	Ion Ampliseq Library Kit plus User Guide	36 – 42	
Library QC qPCR	Ion Universal Library Quantitation Kit	Thermifisher	Ion Ampliseq Library Kit plus User Guide	49 – 50	
6.2.2. Targeted DNA Sequencing					Sequencing: Ion Chef and S5
Library preparation	Ion Ampliseq Library Kit plus, Xpresss barcodes	Thermofisher	Ion Ampliseq Library Kit plus User Guide	25 – 33	
Library QC pPCR	Ion Universal Library Quantitation Kit	Thermofisher	Ion Ampliseq Library Kit plus User Guide	49 – 50	
6.2.3. WES					Sequencing: NovaSeq 6000 (Illumina)
DNA QC	NGS FFPE QC Kit	Agilent	NGS FFPE QC	20 – 28	
Library preparation	SureSelectXT Low Input Target Enrichment System for Illumina, SureSelect human all exon V7	Agilent	SureSelectXT Low Input Target Enrichment System with Dual Indexing	20 – 62	
