# Supplementary material for: Alterations in homologous recombination repair genes in prostate cancer brain metastases
Source: Nat Commun. 2022 May 3;13:2400. doi: 10.1038/s41467-022-30003-5 (PMC9065149; doi:10.1038/s41467-022-30003-5)
Supplement: Supplementary file 3 — Description of Additional Supplementary Files [file 41467_2022_30003_MOESM3_ESM.pdf]

Description of Additional Supplementary Files

File Name: Supplementary Data 1
Description: Regions of interest (ROIs) selected including samples description, methods conducted and pathology assessment (morphology and immunohistochemistry) (n=51 patients)

File Name: Supplementary Data 2
Description: Summary mutation call and TMPRSS2-gene fusions for PCBM-cohort

File Name: Supplementary Data 3
Description: Summary somatic copy number alterations SCNA for PCBM-cohort

File Name: Supplementary Data 4
Description: Summary statistics for the sequenced samples

File Name: Supplementary Data 5
Description: ETS-TMPRSS2 gene fusions sequencing data
