# Supplementary material for: Alterations in homologous recombination repair genes in prostate cancer brain metastases
Source: Nat Commun. 2022 May 3;13:2400. doi: 10.1038/s41467-022-30003-5 (PMC9065149; doi:10.1038/s41467-022-30003-5)
Supplement: Supplementary file 9 — Reporting Summary [file 41467_2022_30003_MOESM9_ESM.pdf]

nature research

Corresponding author(s): Prof. Mark A. Rubin, MD; mark.rubin@dbmr.unibe.ch Salvatore Piscuoglio, PhD s.picuoglio@unibas.ch

Last updated by author(s): 3rd March 2022

Reporting Summary

Nature Research wishes to improve the reproducibility of the work that we publish. This form provides structure for consistency and transparency in reporting. For further information on Nature Research policies, see our Editorial Policies and the Editorial Policy Checklist.

Statistics

For all statistical analyses, confirm that the following items are present in the figure legend, table legend, main text, or Methods section.

n/a	Confirmed	
☐	☒	The exact sample size (n) for each experimental group/condition, given as a discrete number and unit of measurement
☐	☒	A statement on whether measurements were taken from distinct samples or whether the same sample was measured repeatedly
☐	☒	The statistical test(s) used AND whether they are one- or two-sided<br>*Only common tests should be described solely by name; describe more complex techniques in the Methods section.*
☒	☐	A description of all covariates tested
☐	☒	A description of any assumptions or corrections, such as tests of normality and adjustment for multiple comparisons
☐	☒	A full description of the statistical parameters including central tendency (e.g. means) or other basic estimates (e.g. regression coefficient) AND variation (e.g. standard deviation) or associated estimates of uncertainty (e.g. confidence intervals)
☐	☒	For null hypothesis testing, the test statistic (e.g. F, t, r) with confidence intervals, effect sizes, degrees of freedom and P value noted<br>*Give P values as exact values whenever suitable.*
☒	☐	For Bayesian analysis, information on the choice of priors and Markov chain Monte Carlo settings
☒	☐	For hierarchical and complex designs, identification of the appropriate level for tests and full reporting of outcomes
☒	☐	Estimates of effect sizes (e.g. Cohen's d, Pearson's r), indicating how they were calculated

Our web collection on statistics for biologists contains articles on many of the points above.

Software and code

Policy information about availability of computer code

Data collection	Targeted RNA sequencing: Sequencing: Ion Chef and S5.<br>WES sequencing: Sequencing: NovaSeq 6000 (Illumina).
Data analysis	Read alignment: BWA 0.7.17<br>Variant calling: Mutect2 (GATK 4.1.4.1) Strelka2 v2.9.10<br>Copy number analysis: FACETS v0.5.6<br>Clonality: ABSOLUTE V2.0, PyClone 0.13.1<br>Phylogenetic analysis: PhylogicNDT<br><br>R version 3.6.1<br>Mutational Signatures: deconstructSigs v1.8.0, MutationalPatterns v3.3.5<br>Detection of HRD signature: SigMA 1.0.0<br>Organising variant call: maftools v2.0.16<br>Accessing TCGA data: TCGAbiolinks v.2.13.6<br>Generation of figures: ggplot2 v3.3.0, ComplexHeatmap v2.0.0

For manuscripts utilizing custom algorithms or software that are central to the research but not yet described in published literature, software must be made available to editors and reviewers. We strongly encourage code deposition in a community repository (e.g. GitHub). See the Nature Research guidelines for submitting code & software for further information.

Data

Policy information about availability of data

All manuscripts must include a data availability statement. This statement should provide the following information, where applicable:

- Accession codes, unique identifiers, or web links for publicly available datasets
- A list of figures that have associated raw data
- A description of any restrictions on data availability

Every analyzed sample got an unique identifier (Supplemental data SD6).
Data from comparison cohorts (CRPC500 and TCGA) can be found publicly available under: https://www.cbioportal.org
Aligned whole-exome sequencing data are available from the European Genome-phenome Archive (EGAS00001005091) [https://ega-archive.org/studies/EGAS00001005091]

Field-specific reporting

Please select the one below that is the best fit for your research. If you are not sure, read the appropriate sections before making your selection.

- [x] Life sciences
- [ ] Behavioural & social sciences
- [ ] Ecological, evolutionary & environmental sciences

For a reference copy of the document with all sections, see nature.com/documents/nr-reporting-summary-flat.pdf

Life sciences study design

All studies must disclose on these points even when the disclosure is negative.

Sample size	We included 51 patients with metastatic prostate cancer (CNS -brain/spinal cord- and meningeal) and with matched primary tumors in 20 cases. From 32/51 patients we performed WES analyses in normal tissue samples. This was a multi-centre, international study, representing the largest cohort of prostate cancer brain metastases studied to date. Further samples were not available.
Data exclusions	None
Replication	No replication was performed in obtaining WES or targeted RNA data. Multiregion sequencing was performed on tumours from a cohort of patients. Technical replicates are therefore not needed.
Randomization	Not relevant for this study, no prognostic or predictive analyses were performed. Study was a direct comparison of prostate cancer brain metastases, with primary PCa, and metastases from other sites.
Blinding	Not relevant for this study, no prognostic or predictive analyses were performed. Study was a direct comparison of prostate cancer brain metastases, with primary PCa, and metastases from other sites.

Reporting for specific materials, systems and methods

We require information from authors about some types of materials, experimental systems and methods used in many studies. Here, indicate whether each material, system or method listed is relevant to your study. If you are not sure if a list item applies to your research, read the appropriate section before selecting a response.

Materials & experimental systems

n/a	Involved in the study	
☐	☒	Antibodies
☒	☐	Eukaryotic cell lines
☒	☐	Palaeontology and archaeology
☒	☐	Animals and other organisms
☐	☒	Human research participants
☒	☐	Clinical data
☒	☐	Dual use research of concern

Methods

n/a	Involved in the study	
☒	☐	ChIP-seq
☒	☐	Flow cytometry
☒	☐	MRI-based neuroimaging

Antibodies

Antibodies used	1. p53 (clone DO-7; Dako-Agilent); M7001; RRID:AB_2206626; 1:800<br>2. PTEN (clone 6H2.1; Cascade Bioscience); ABM-2052; RRID:AB_2335636; 1:400<br>3. ERG (clone EP111; Dako-Agilent); M73149; 1:50<br>4. Chromogranin-A (clone DAK-A3; Dako-Agilent); M086901; RRID:AB_2081135; 1:1600

5. Synaptophysin (clone 27G12, BioSystems); NCL-L-SYNAP-299; RRID:AB_442136; 1:100
6. PSA (polyclonal; Dako-Agilent); A0562; RRID:AB_2768590; 1:4000
7. CK5/6 (clone D5/16 B4; Merck); MAB1620; RRID:AB_94292; 1:4000
8. p63 (clone 7JUL; BioSystems); NCL-L-p63; 1:40

Validation

All antibodies used were previously validated for human diagnostic purposes in the accredited Institute of Pathology (Bern, Switzerland). RRID provided if available.

Human research participants

Policy information about studies involving human research participants

Population characteristics

We collected retrospectively FFPE from patients who fulfil the criteria as defined: male gender; age ≥ 18 years; histologically confirmed CNS/meningeal metastasis from prostate cancer; available archival biopsy. Additionally, if available, we collected archival available FFPE tissue from the matched primary prostate cancer. Average age of the patients at Diagnosis of PCBM was 71 years old (Range: 48-81 years old)

Recruitment

Tumor samples were collected from Pathology Departments in University and Cantonal Hospitals across Switzerland (Institute of Pathology, Bern/ Institute of Neuropathology, Zurich/ Institute of Pathology, Aarau).

Ethics oversight

All analyses were carried out in accordance with protocols approved by the Ethical Committee Bern (Project ID: 2019 – 00328). No participant compensation was applied for the current study.

Note that full information on the approval of the study protocol must also be provided in the manuscript.
